# Intestinal ‘Infant-Type’ Bifidobacteria Mediate Immune System Development in the First 1000 Days of Life

**DOI:** 10.3390/nu14071498

**Published:** 2022-04-02

**Authors:** Chunxiu Lin, Yugui Lin, Heng Zhang, Gang Wang, Jianxin Zhao, Hao Zhang, Wei Chen

**Affiliations:** 1 State Key Laboratory of Food Science and Technology, Jiangnan University, Wuxi 214122, China; 7200112017@stu.jiangnan.edu.cn (C.L.); wanggang@jiangnan.edu.cn (G.W.); zhaojianxin@jiangnan.edu.cn (J.Z.); zhanghao61@jiangnan.edu.cn (H.Z.); 2 Zhongshan Bo’ai Hospital, Southern Medical University, Zhongshan 528400, China; boai18324180870@126.com; 3 Wuxi Maternity and Child Health Care Hospital, Wuxi 214002, China; 4 School of Food Science and Technology, Jiangnan University, Wuxi 214122, China; 5 (Yangzhou) Institute of Food Biotechnology, Jiangnan University, Yangzhou 225004, China; 6 National Engineering Center of Functional Food, Jiangnan University, Wuxi 214122, China; 7 Wuxi Translational Medicine Research Center, Jiangsu Translational Medicine Research Institute Wuxi Branch, Wuxi 214122, China

**Keywords:** infant-type bifidobacteria, neonatal immune systems, human milk oligosaccharides, the first 1000 days of life, immune tolerance, intestinal inflammation, intestinal microecology, immune-mediated disorders

## Abstract

Immune system maturation begins early in life, but few studies have examined how early-life gut microbiota colonization educates the neonatal immune system. Bifidobacteria predominate in the intestines of breastfed infants and metabolize human milk oligosaccharides. This glycolytic activity alters the intestinal microenvironment and consequently stimulates immune system maturation at the neonatal stage. However, few studies have provided mechanistic insights into the contribution of ‘infant-type’ *Bifidobacterium* species, especially via metabolites such as short-chain fatty acids. In this review, we highlight the first 1000 days of life, which provide a window of opportunity for infant-type bifidobacteria to educate the neonatal immune system. Furthermore, we discuss the instrumental role of infant-type bifidobacteria in the education of the neonatal immune system by inducing immune tolerance and suppressing intestinal inflammation, and the potential underlying mechanism of this immune effect in the first 1000 days of life. We also summarize recent research that suggests the administration of infant-type bifidobacteria helps to modify the intestinal microecology and prevent the progress of immune-mediated disorders.

## 1. Introduction

**Figure 1:**
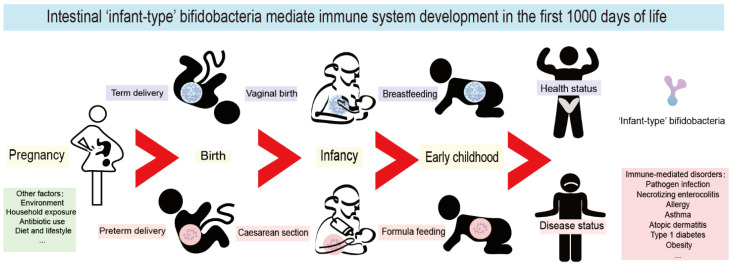
Intestinal infant-type bifidobacteria mediate immune system development in the first 1000 days of life. The mode of delivery and feeding exert the most pronounced roles in the colonization of infant-type bifidobacteria in the first 1000 days. Pregnancy, environment, household exposure, antibiotic use, diet, and lifestyle all have a far-reaching impact on the composition of infant intestinal flora. Interfering with the colonization of infant-type bifidobacteria in early life leads to long-term and far-reaching health consequences, especially mediating the occurrence and development of a variety of immune-mediated disorders, including pathogen infection, necrotizing enterocolitis (NEC), allergy, asthma, atopic dermatitis, type 1 diabetes mellitus (T1D) and obesity.

During the first 1000 days of life, immune system maturation and gut microbiota establishment occur concurrently, and this phenomenon has become one of the most exciting areas of immunological research. Due to potential microbiological contamination, it has not yet been possible to determine the exact bacterial composition of prenatal meconium [1,2,3]. However, it is well known that extensive microbial colonization begins rapidly after delivery [4]. In the first 1000 days of life, the gut microbiota of healthy breastfed infants is typically dominated by ‘infant-type’ bifidobacteria, including *Bifidobacterium longum* subsp. *infantis* (*B. longum* subsp. *infantis*), *B. bifidum*, *B. breve* and *B. longum* subsp. *longum* [5,6]. It is increasingly believed that the first 1000 days of life provide a critical window of opportunity for the shaping of the early immune system; thus, it might influence short-term and long-term host health by infant-type bifidobacterial colonization (Figure 1).

To utilize host-derived nutrients, intestinal infant-type bifidobacteria have evolved a series of complex genetic pathways to metabolize human milk oligosaccharides (HMOs) [7,8]. This glycolytic activity leads to an anaerobic and acidic intestinal environment that stimulates immune development, controls pathogens, and affects the development of many organs (e.g., the liver and the brain) [9,10,11]. Moreover, a plethora of studies has provided evidence to support the association of the reduced abundance of infant-type bifidobacterial species with immune disorders in children or adults, such as pathogen infection, necrotizing enterocolitis (NEC), allergy, asthma, atopic dermatitis, type 1 diabetes mellitus (T1D), and obesity [12,13]. In addition, impaired microbiota development has been observed in preterm infants, infants delivered by cesarean section, and infants exposed to antibiotics in early life, which is often characterized by a reduced abundance of infant-type *Bifidobacterium* species [14,15]. Infant-type bifidobacteria are thus widely considered critical for the healthy development of the intestinal microbiota and immune system in the first 1000 days of life. Therefore, gut microbiome manipulation strategies are receiving increasing attention, especially those involving the generation or supplementation of infant-type bifidobacteria to help restore immune system development [9,16,17,18,19].

The purpose of this review is to highlight the core immunomodulatory role of infant-type bifidobacteria as keystone intestinal flora in the first 1000 days of life. The review also provides mechanistic insights into the application of infant-type *Bifidobacterium* species as a promising microbiome regulator to lay the foundation for intestinal health and immune homeostasis in children.

## 2. What Are the ‘Infant-Type’ *Bifidobacterium* Species and the Effect of Cross-Feeding?

The bifidobacteria isolated from the human gastrointestinal tract can be divided into two major categories: ‘adult-type’ *Bifidobacterium* species derived from the adult feces (including *B. adolescentis*, *B. catenulatum* and *B. pseudocatenulatum*), and infant-type *Bifidobacterium* species, encompassing other bifidobacteria that naturally inhabit the infant gut (including *B. longum* subsp. *infantis*, *B. bifidum*, *B. breve* and *B. longum* subsp. *longum*) [5,6,20]. *B. longum* subsp. *longum* is predominant in infant, adult, and elderly intestines [21]. Of note, *B. longum* subsp. *longum* is more common in formula-fed infants, while *B. longum* subsp. *infantis* is more common in breastfed infants [22]. Infant-type *Bifidobacterium* species possess genetic and enzymatic toolsets that are specific for HMO utilization; this confers upon them a growth advantage over other bacterial species, such as adult-type bifidobacteria and pathogens, enabling them to thrive in the intestines of infants in early life [7,8].

Notably, the utilization of HMOs by bifidobacteria shows species-specific differences. *B. longum* subsp. *infantis* possesses all the critical genes required for the complete internal degradation of HMOs and has been found to consume HMOs over other carbohydrates preferentially [8,23]. *B. bifidum* and *B. breve* are endowed with only very limited capabilities in utilizing HMOs [23]. It is evident that *B. longum* subsp. *infantis* is the most effective consumer of HMOs, and *B. bifidum* and *B. breve* can also partially consume HMOs, which are commonly found in breastfed infants [23]. However, *B. longum* subsp. *longum* and *B. adolescentis* mainly consume plant-derived, animal-derived, and host-metabolized carbohydrates, which results in their predominance in adults [8].

Moreover, certain *Bifidobacterium* species’ abundance in infants is associated with feeding patterns. HMOs are abundant in breast milk but are not or are very uncommon in infant formula [24]. Nowadays, HMOs are emerging in infant formulae to mimic natural human milk [25]. However, the infant formulae currently on the market, even the most premium ones, cannot provide the full range of HMOs [23]. More than 200 HMOs have been identified in breast milk, while only 2′-fucosyllactose (2′FL) and lacto-N-neotetraose (LNnT) are the two major HMOs applied to high-end and ultra-high-end infant formulae [25,26]. In addition, for many valid reasons, not all bottle-fed babies are fortunate enough to consume formula supplemented with HMOs [27]. As a result, most formula-fed infants are routinely not receiving adequate and abundant amounts of HMOs, which causes a decrease in the abundance of infant-type bifidobacteria.

Furthermore, the bifidobacterial population in the infant’s gut is composed of a co-group of multiple *Bifidobacterium* strains, rather than one strain dominating and competing to the exclusion of all others [28]. On the one hand, the cross-feeding effect among bifidobacterial species/strains is associated with the ability to thrive in HMOs of multiple *Bifidobacterium* members in the infant’s gut [29]. According to a study, the total abundance of *Bifidobacteriaceae* became very high when the abundance of *B. bifidum* exceeded 10% of the total microbiota in the feces of breastfed infants, suggesting the cross-feeding of HMO degraders in the bifidobacterial taxa [30]. It was found that *B. bifidum* SC555, as a fucose provider by hydrolyzing 2′FL, fulfilled the cross-feeding function of *B. breve* [31]. Although *B. breve* has no direct access to HMOs as a carbon source, host-derived monosaccharides/oligosaccharides could be vigorously consumed by the hydrolytic activity of other bifidobacteria [32]. In this context, the moderate growth of *B. breve* in total HMO and good growth in lacto-N-tetraose (LNT) and LNnT were observed [33]. As a result, *B. breve* was isolated from the feces of breastfed infants at a high frequency. Similarly, *B. pseudocatenulatum* and *B. kashiwanohense* can barely consume HMOs and require further growth based on the degradation products of other bifidobacterial HMO utilizers [32]. On the other hand, certain bifidobacterial taxa cooperate with non-bifidobacterial taxa (including HMO consumers and non-HMO consumers) to maximize the nutrient consumption of HMOs, thus contributing to increased bifidobacterial diversity and dominance-gaining [29]. Considering the ability to digest HMOs, recent studies have confirmed that *Lactobacillus*, *Bacteroides* and *Fragilis* might also dominate the gut microbiota of infants to some extent [34]. Although other genera of neonatal intestinal microbiota (such as *Clostridium*, *Enterococcus*, *Escherichia*, *Staphylococcus,* and *Streptococcus*) cannot degrade HMOs on their own, partial decomposition products or fermentation end-products might be produced in combination with *Bifidobacterium* and/or *Bacteroides* [35,36]. Taken as a whole, this highlights the cooperation of the bacterial community in the neonatal intestine to maximize the utilization of HMOs, so as to maintain the intestinal immune balance of newborns.

This suggests a clear evolutionary link between infant-type bifidobacteria, neonatal immune systems and HMO metabolism. Overall, infant-type *Bifidobacterium* species are well adapted to the infant gut and efficiently consume HMOs, and their presence influences both immediate and long-term health outcomes.

## 3. Establishment and Evolution of Infant-Type Bifidobacteria

When it comes to the establishment and evolution of infant-type bifidobacteria, we naturally think about where it comes from. Is it prenatal or postpartum? For more than a century, it was believed that the fetal environment in the womb is sterile, and that the fetal immune system is immature and inactive [37,38]. Recent discoveries made via the next-generation sequencing of the placenta samples, amniotic fluid, meconium, and fetal tissue have challenged this sterile uterus dogma [37]. However, while the critical roles of bifidobacteria as commensals and symbionts in modulating the immune system are understood, research on whether prenatal mother–fetal bifidobacterial exchange occurs has been inconclusive [1,2,3]. This area remains largely unexplored, despite the finding that maternal microorganisms can influence fetal immune development. The initial antibodies in the fetus appear due to transplacental circulation during pregnancy and play a vital role in protecting the newborn from infection before and after birth [39]. Antibodies are also transferred to infants through their mother’s milk, especially immunoglobulin (Ig) A, which protects preterm infants from NEC [40]. In addition, maternal microorganisms produce myriad metabolites with a wide range of immunomodulatory functions, some of which are transmitted from the mother to the child during pregnancy and lactation [39,41]. The most common metabolites that are transferred are short-chain fatty acids (SCFAs), which reduce cytokine storms by promoting the development of regulatory T cells (Tregs) [39]. The above findings suggest that before the birth of an infant, the maternal microbiome provides a ‘distance’ education that stimulates, to some extent, the development of the infant’s immunity.

Regardless of intrauterine exposure, a wide variety of bacteria infiltrate the neonatal gastrointestinal tract, actively or passively, via the mouth and nose (through breathing, feeding, crying) and anus (defecating), within a few hours after delivery. Maternal microbiome acquisition in early life might occur through several mechanisms, including early exposure to the vaginal and fecal microflora of the mother, the transmission of maternal skin-related microorganisms, and the transfer of microorganisms through breast milk [14,15]. Birth via cesarean section is known to interrupt the vertical transmission of the maternal gut and vaginal microbiota to infants. Most of the intestinal bacteria of vaginally delivered infants originate from the mother’s gut and/or vagina, whereas those of cesarean-delivered infants mainly originate from the mother’s oral cavity and skin and from the hospital environment, all of which have a higher abundance of *Enterococcus*, *Enterobacter* and *Klebsiella* species and a lower abundance of bifidobacteria [14,15]. Moreover, compared with vaginal delivery, cesarean delivery might delay breastfeeding initiation and reduce breast milk supply and receptivity to breastfeeding, which might further compromise the development of the infant microbiome in early life [42].

The infant gut microbiota initially shows a low diversity and then increases with early development. Immediately after birth, the infant’s gut is first colonized by facultative anaerobes, such as *Staphylococcus* spp., *Streptococcus* spp., *Enterobacter* spp., and other members of the Enterobacteriaceae family. These anaerobes gradually deplete intestinal oxygen and reduce intestinal oxidation-reduction potential within 48 h of birth, which facilitates the colonization of absolute anaerobic bacteria such as *Bifidobacterium*, *Clostridia,* and *Bacteroides* [43,44]. Approximately one week after delivery, *Bifidobacterium* is the most predominant genus, and represents a significant proportion of the gut microbiota of a healthy infant throughout the breastfeeding period, accounting for 40–80% of the total intestinal microbiota [45,46]. The colonization of infant-type bifidobacterial strains in the intestinal tract of infants is not deterministic but is instead strongly driven by early-life nutrition, especially by breast milk [47]. The third-largest solid component in breast milk, HMOs have a bifidogenic effect on the infant’s microbiota by supporting the growth of intestinal infant-type bifidobacteria [48]. In addition, breastfed babies also have diverse gut microbiotas; these depend on the mother’s nutrition during pregnancy, as well as physical fitness, etc. For example, the high-fat diet of breastfeeding mothers is linked to gut flora dysbiosis in their infants [49]. Before weaning, *Bifidobacterium* in the infant’s intestine gradually decreases due to competition from *Bacteroides* [34]. After weaning, bifidobacteria decrease, together with *Clostridium perfringens* and *Clostridium difficile*, while the abundance of other strictly anaerobic *Clostridium* increases [50]. Subsequently, with the increase in the number and variety of supplementary foods, the bifidobacterial composition of the intestinal flora of infants begins to gradually change from infant-type to adult-type, becoming progressively more complex during the first year of life and approaching that of an adult at approximately three years of age [51,52]. Aside from the mode of delivery and type of diet, the two most important factors shaping the intestinal flora of newborns, pregnancy (term delivery vs. preterm delivery), environment (rural vs. urban), household exposure (e.g., siblings and furry pets), antibiotic use and lifestyle are known to have far-reaching effects on the abundance and species composition of bifidobacteria. These factors are investigated in detail in the literature [53].

## 4. The First 1000 Days of Life Are a Key Window of Opportunity for Immune System Maturation

As shown in Figure 1, the first 1000 days of life represent the initial period from the formation of the fertilized ovum and the intrauterine growth of the fetus to the second year after birth. The presence of infant-type bifidobacteria strains in newborns is considered to be a sign of healthy microbiota development and immune system maturation during the first 1000 days of life. Mounting evidence shows this period to be a critical window of opportunity for the colonization and establishment of the first intestinal microorganisms and the development and maturation of the immune system, both of which influence host health in adolescence and even in adulthood [4].

In 2008, Victora et al. [54] reported the effect of maternal and infant malnutrition on infant health. They found that the critical foundations for lifetime health outcomes are laid down from the fetal period to 2 years after birth, thus proposing the concept of the first 1000 days of life [54]. Subsequently, in 2013, Balbus et al. [55] noted that more attention should be paid to early postpartum interventions, nutrition optimization and the prevention of exposure to toxins to reduce the prevalence of non-communicable diseases. Since then, there has been an increasing research focus on the first 1000 days of life as the starting point for a healthy future.

Nowadays, breastfeeding alone is insufficient to protect infants against pathogens and develop a healthy immune system, suggesting the need to develop infant-type bifidobacteria supplements. Recent studies have reported that breastfed infants are increasingly found to have manifestations of intestinal microbiota dysfunction [56,57]. The abundance of bifidobacteria in breastfed infants in resource-rich countries has decreased with the generations over the past 100 years, accompanied by increased levels of intestinal pathogens and fecal pH [56]. This might be attributed to the individual differences of cofactors in breast milk, the use of antibiotics, the frequency of cesarean sections and the increase in potential pathogens [57]. More worryingly, not all babies are fortunate enough to be fed with their mother’s breast milk for many legitimate reasons, but formulae are fully or partially HMO-free. A growing number of studies have shown that infants should be fed a diet that supplements infant-type bifidobacteria early to maintain intestinal health, inhibit the abundance of harmful bacteria, and reduce the risk of allergies and autoimmune diseases [17,57,58].

## 5. Infant-Type Bifidobacteria Affect the Establishment of Immunity in Early Life

As mentioned, the first 1000 days of life provide a critical window of opportunity for immune system maturation, including the establishment of adaptive immunity and immune tolerance. During this period, the intestinal microbiome of the fetus and the infant undergo substantial development: from being in a near-sterile environment in the womb to being rapidly colonized by a large number of microbes at birth. There is mounting evidence that intestinal infant-type bifidobacteria are ideal probiotics for infants and are vital to early-life immunological development [9,59,60,61]. Herein, we discuss what is known about the role of intestinal infant-type bifidobacteria in establishing immune function in the early stages of life.

### 5.1. Infant-Type Bifidobacteria Occupy Intestinal Ecological Sites

**Figure 2:**
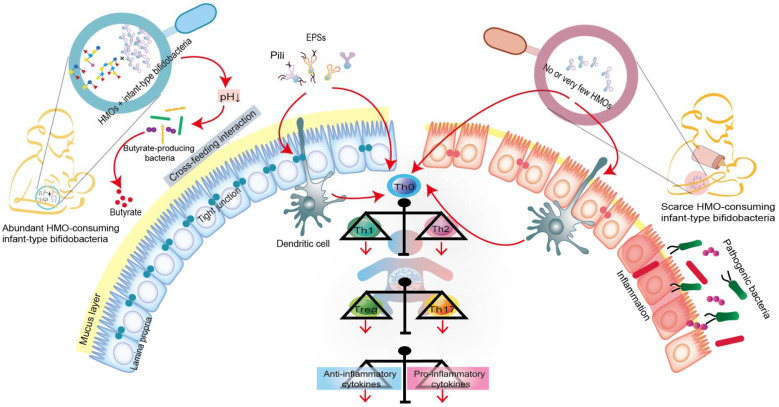
Co-evolution of intestinal infant-type bifidobacteria and HMOs-mediated immune system imprinting early in life. Breastfed infants are rich in intestinal infant-type bifidobacteria, which is significantly correlated with the increase in anti-inflammatory cytokines. In contrast, infants without breastfeeding have lower or no intestinal infant-type bifidobacteria, resulting in increased levels of pro-inflammatory cytokines. Although the exact mechanism is still elusive, infant-type bifidobacteria are widely considered to successfully modify the intestinal microecology in the early stages of life and prevent the progress of immune-mediated diseases, at least, by producing short-chain fatty acids (SAFAs), encoding extracellular structures (exopolysaccharides (EPSs) or/and pili), promoting cross-feeding effects, skewing T cell polarization, and promoting the expression of anti-inflammatory cytokines.

Humans cannot digest HMOs due to the lack of the necessary enzyme (glucosidase); this provides a selective nutritional advantage for the growth of beneficial bacteria that specifically consume HMOs [9,62]. Moreover, intestinal infant-type bifidobacteria, the main drivers of HMO metabolism, have evolved complex genetic pathways to metabolize the different glycans in human milk [7,8]. Intestinal infant-type bifidobacteria, therefore, have a competitive advantage in colonization over other gastrointestinal bacteria. In addition, the saccharolytic activities of bifidobacteria generate an anaerobic and acidic intestinal environment that protects the intestine from pathogenic infection. For example, compared to the stool samples of breastfed infants who received no supplementation, the stool samples from breastfed infants who were supplemented with *B. longum* subsp. *infantis* EVC001 exhibited a decrease in the abundance of Gram-negative *Proteobacteria* and *Bacteroides* and a four-fold decrease in the levels of endotoxin [17]. This indicates that infant-type bifidobacteria play a critical role in mediating immunity by occupying intestinal ecological sites and preventing pathogens from invading the intestine (Figure 2).

### 5.2. Infant-Type Bifidobacteria Facilitate Breast-Milk Metabolism

Infant-type bifidobacteria can affect host physiology in multiple ways, such as by regulating breast-milk metabolism. As mentioned above, it is now widely accepted that the co-evolution of infant-type bifidobacteria and the host immune system, mediated by HMOs, profoundly shapes early intestinal flora colonization and strongly influences the neonatal immune system. However, little is known about infant-type bifidobacterial metabolites, such as SCFAs, that mediate host-microbe interactions [63].

We focused on three common short-chain fatty acids (acetic acid, propionic acid and butyric acid) and lactic acid (also produced by gut bacteria and playing an important role in gut health) to learn how they contribute to gut health and immune development early in life. Infant-type bifidobacteria convert HMOs into acidic end-products (acetic acid and lactic acid) that affect fecal pH, reduce intestinal permeability, and increase the stability of tight junction proteins, all of which are characteristic of some intestinal diseases, such as NEC (Figure 2) [10,11,63]. Moreover, the acetic acid produced by infant-type bifidobacteria indirectly stimulates the growth, function and immune response of butyric acid-producing microorganisms through a mutually beneficial cross-feeding interaction (Figure 2) [64]. Butyric acid is the preferred fuel for intestinal epithelial cells, and its production further promotes the maintenance of intestinal barrier function by increasing mucin production and improving tight junction integrity [63,65]. In addition to their role in the intestinal tract, SCFAs produced by intestinal infant-type bifidobacteria can affect host physiology outside of the intestine, e.g., in the lung, brain, liver and adipose tissue [10,11]. For example, acetic acid, as the most abundant SCFA in the peripheral circulation, plays a direct role in central appetite regulation by crossing the blood-brain barrier and being absorbed by the brain, resulting in appetite suppression and hypothalamic neuron activation [10]. Although the concentrations of propionic acid and butyric acid are low in peripheral circulation, they indirectly affect the peripheral organs by activating hormone production and the nervous system [11].

Recent studies have also demonstrated the importance of aromatic amino acids, a type of metabolite produced by infant-type bifidobacteria, in early life [9,59]. Infant-type bifidobacteria produce large amounts of aromatic lactic acids, such as tryptophan-derived indole-3-lactic acid (ILA), in the intestinal tract of infants, through the action of aromatic lactate dehydrogenase [59]. Recently, ILA was shown to bind to both the aryl hydrocarbon receptor and hydrocarboxylic acid receptor 3, regulate the response of monocytes to lipopolysaccharides, and block the transcription of inflammatory cytokines such as interleukin (IL)-8 [59]. ILA is also an anti-inflammatory molecule that can promote the development of immature intestinal epithelial cells [59]. Similarly, Henrick et al. observed that ILA produced by *B. longum* subsp. *infantis* silences the T helper cell 2 (Th2) and Th17 immune responses that are required to induce immune tolerance and inhibit intestinal inflammation during early life [9]. These new findings suggest that tryptophan metabolic pathways, particularly the ILA pathway, might be crucial for early immune development mediated by infant-type bifidobacteria, which maintains the inflammatory/anti-inflammatory balance of neonatal immunity, blocks the production of inflammatory cytokines and mitigates inflammatory damage.

### 5.3. Infant-Type Bifidobacteria Promote Immune Development and Prime the Anti-Inflammatory Gene Pool

As mentioned above, the immune system is immature in the early stages of life. Intestinal infant-type bifidobacteria represent one of the earliest antigens to activate the host defense mechanism, which helps to promote immune development and prime the anti-inflammatory gene pool (Figure 2). Recent studies have shown that infant-type bifidobacteria present different cell surface structures, such as exopolysaccharides (EPSs) and pili/fimbriae, allowing bifidobacteria to colonize the infant’s gut, adhere to intestinal cells and participate in the development of the host immune system [66,67]. Consequently, the administration of EPSs from *B. breve* UCC2003 might serve as a new strategy for promoting infant health [68]. In addition, EPSs produced by *B. longum* subsp. *longum* 35624 plays an essential role in inhibiting the pro-inflammatory response of the host (especially the local Th17 response), thus preventing inflammatory diseases [69]. Pili represent a fundamental cell-surface structure considered to be crucial in the interaction between *Bifidobacterium* and its host, and *B. bifidum* PRL2010 is equipped with the most functionally characteristic sortase-dependent pili [70,71].

Furthermore, the colonized intestinal microflora can trigger various immune responses by activating T cells and B cells in the intestinal mucosa. In the neonatal period, inflammatory responses are actively suppressed. This phenomenon occurs because neonatal T cells have an internal mechanism wherein the immune system develops toward the characteristics of sensitizing Th2 cytokines, while Th1 cell proliferation and interferon-γ production are inhibited (Figure 2) [38,72]. A recent study showed that the absence of bifidobacteria in the gut flora of newborns leads to the activation of innate and adaptive immunity, which increases the populations of neutrophils, basophils, plasmablasts and memory CD8^+^ T cells [9]. Additionally, intestinal infant-type bifidobacteria affect the intestinal Th cell response, especially that of Th0 and Th17 cells [9]. In particular, these bifidobacteria stimulate the early immune response for defense while avoiding the overactivation of Th2 and Th17 responses [9]. This study found that the increase in infant-type bifidobacteria abundance is positively associated with a decrease in pro-inflammatory markers (such as memory Tregs and pro-inflammatory monocytes) and an increase in inflammatory markers (such as Tregs, IL-10 and IL-27) [9]. Another study showed that the growth of HMO-consuming *B. longum* subsp. *infantis* stimulates the activity of the intestinal epithelium via T cells [61]. Specifically, *B. longum* subsp. *infantis,* grown on HMO, increased the expression of the anti-inflammatory cytokine IL-10 in Caco-2 cells and the expression of junctional adhesion molecules and occludin in Caco-2 cells and HT-29 cells [61]. Furthermore, the infant-type bifidobacteria might affect T cells directly or indirectly, via dendritic cells [66,73]. Recent research provides new evidence of the crucial role of breast milk in promoting neonatal *Bifidobacterium* colonization and B cell activation [74]. It shows that core-fucosylated *N*-glycans in the mother’s milk selectively promote the colonization of bifidobacteria in infants, whereas their absence from the mother’s milk reduces the proportion of spleen CD19^+^ CD69^+^ B cells in offspring mice. In vitro studies also found that the L-fucose metabolites, lactic acid and 1,2-propanediol promote B cell activation through the signaling pathway mediated by B cell receptors [74].

### 5.4. The Potential Role of Infant-Type Bifidobacteria in Early Neuroimmune Development

Recent studies have emphasized the potential role of intestinal infant-type bifidobacteria in neuroregulation in early life [75,76]. A growing body of evidence supports that the gut-brain connection already exists at birth and enables the gut microbiome to interact with and affect the central nervous system [75,76]. The accumulated data show that neonatal intestinal microbial colonization activates the immune system, sends signals to the central nervous system through the afferent vagus nerve, and produces microbial metabolites (SCFAs, such as acetic acid) that can directly or indirectly affect brain function [75]. Thus, the intestinal microbiota that colonizes infants during their early life shapes the steady-state development and balance of postnatal neural connection [75]. Intestinal infant-type bifidobacteria might also shape host neural circuits by regulating synaptic gene expression and microglial functionality during postnatal development [75]. Currently, however, there is limited information on the mechanism by which gut microbes communicate with the host brain. Further studies are warranted to further clarify the effects of infant-type bifidobacteria on brain function and the underlying mechanism of their action in newborns.

In short, intestinal infant-type bifidobacteria trigger immunomodulatory responses through multiple mechanisms to maintain host health (Figure 2). For example, bifidobacteria occupy the microbial nutritional niche in the gut, release metabolic by-products of HMOs either directly or indirectly (cross-feeding interaction), present extracellular structures (such as EPSs and pili) that interact with host cells, and inhibit inflammatory responses, including intestinal Th cell responses and B cell-induction responses. Observational studies have confirmed a link between the loss of infant-type bifidobacteria and early intestinal inflammation, although little is known about the underlying mechanisms [9,59,76,77].

## 6. Infant-Type Bifidobacteria Supplementation Is a Promising Strategy for Immune-Mediated Disorders

In fact, some immune-mediated diseases are associated with gut dysbiosis in infancy, often as a result of absent or insufficient breastfeeding. Indeed, breastfeeding is considered to be the best form of infant feeding, which has the potential to overcome immune-mediated disorders. However, over the past 100 years, breastfed infants have experienced a disturbing upheaval in the gut microbiota, with a reduction in fecal bifidobacteria and a dramatic rise in pH to 6.5 [56]. Accumulated evidence suggested that supplementation with *B*. *longum* subsp. *infantis* was recommended for breastfed infants to promote healthy intestinal development [17,57,58]. Therefore, it is emphasized here that infant-type bifidobacteria supplementation is a promising strategy to avoid immune-mediated disorders, even in breast milk-fed babies. Given that the intestinal microbiome is modifiable, optimizing its composition in early life might be a potential approach for treating immune-mediated disease. Here, we review the latest studies that have investigated the critical role of the early-stage microbiota in immune-mediated disease progression. We also discuss whether infant-type bifidobacteria supplementation could alter the composition of the early intestinal microbiota and thereby prevent the progression of immune-mediated disease (Table 1).

### 6.1. Effects of Infant-Type Bifidobacteria on Pathogen Infection and NEC

The neonatal gut is immature and is susceptible to colonization by pathogenic bacteria; therefore, gut colonization by healthy microflora in early life helps to protect infants from pathogenic infection. Rotavirus is a common pathogen that can cause severe diarrhea and fatal dehydration in infants and young children. Studies have shown that the administration of *B. breve* M-16V to Lewis rats 2–14 days after birth can effectively reduce the severity and incidence of infectious diarrhea caused by rotavirus [79]. In addition, it has been suggested that the administration of infant-type bifidobacteria could prevent and treat rotaviral infection [80,81]. Moreover, early gut colonization by *Bifidobacterium*, especially *B. longum* subsp. *infantis,* enhances the response to early vaccination: the average abundance of bifidobacteria in early infancy was positively correlated with the CD4 T-cell responses to *Bacillus* Calmette-Guérin, tetanus toxoid and hepatitis B virus at 15 weeks [73]. Interestingly, the positive response of CD4 T cells to *Bacillus* Calmette-Guérin and tetanus toxoid was found to be maintained for over two years [73]. Moreover, the mean abundance of bifidobacteria was positively correlated with plasma tetanus toxoid-specific IgG concentrations and stool polio-specific IgA concentrations at 2 years [73]. A similar result was obtained in the dominant subspecies, *B. longum* subsp. *infantis* [73].

NEC is one of the most common and severe gastrointestinal tract diseases; it has high morbidity and mortality rates and mainly occurs in premature infants. In NEC, excessive inflammation is caused by the high immunoreactivity of the intestine and affects distant organs such as the brain, liver and lungs [86,87]. Infants who recover from NEC have increased risks of developing short bowel syndrome, parenteral nutrition-related liver disease, pulmonary hypertension, microcephaly and severe neurodevelopmental delays [86,87]. Many microbiological and molecular biological studies have been conducted on the intestines of infants with NEC, but the cause and mechanism of NEC remain incompletely understood. According to the hypothesis proposed by Claud and Walker, the occurrence of NEC in premature infants results from the synergistic effects of multiple risk factors, with inappropriate initial microbial colonization of the gut being one of the most critical risk factors [88]. Specifically, it is thought that inappropriate initial microbial colonization exacerbates the risk of inflammation in the intestine, which is also a crucial neonatal risk factor for NEC [89]. Studies have confirmed the positive role of infant-type bifidobacteria in preventing and treating NEC. Hoyos was the first to describe infant-type bifidobacteria as probiotics that had therapeutic effects on NEC [78]. In 1999, she found that the oral administration of prophylactic *B. longum* subsp. *infantis* and *Lactobacillus acidophilus* (at a dose of 2.5 × 10^8^ CFU of each organism) for one year decreased the incidence of and mortality due to NEC in 1237 neonates in an intensive-care unit [78]. Subsequent follow-up studies have found that the relative risk of NEC reduced significantly in infants on probiotic treatment regimens that consisted of infant-type bifidobacteria, although the optimal strains, treatment durations and dosages have not yet been determined [16,82,83,84,85]. The administration of antibiotics to preterm and low-birthweight infants disrupts the gut colonization pattern of bifidobacteria, thereby reducing infants’ intestinal resistance to pathogenic bacteria and increasing their risk of NEC [90,91,92]. Therefore, antibiotic treatment for infants should be avoided unless absolutely essential. In summary, changing the composition of early gut flora, especially by increasing the proportion of infant-type bifidobacteria, is a promising strategy for reducing the risk of early life-threatening infections in infants.

### 6.2. Effects of Infant-Type Bifidobacteria on Allergic Diseases

The prevalence of allergic diseases has been increasing globally in recent decades, especially in low- and middle-income countries, and these diseases currently affect approximately 25% of the world’s population [93,94]. In high-income countries, deficiencies in intestinal infant-type bifidobacteria are common in newborns and are likely to contribute to an increase in the incidence of allergic diseases [19,95]. In contrast, in developing countries, children with high rates of *B. longum* subsp. *infantis* colonization were found to be at low risk of atopic diseases [19]. Although the pathogenesis of allergic diseases might be multifactorial, many studies have provided evidence suggesting that inappropriate early intestinal flora colonization is the main reason for the disruption of normal postnatal immune regulation. For example, an imbalance in the composition of intestinal flora leading to a low abundance of bifidobacteria in newborns predates the development of atopic diseases [96]. In addition, compared with newborns, in older children, there is a less consistent association between the composition of the gut microbiota and the development of allergic diseases or asthma [97]. Hence, it appears that disturbances in the early gut microbiota, or dysbiosis, might drive the occurrence of allergic diseases, which might last a lifetime. Regulating the early intestinal microbiota through the administration of infant-type bifidobacteria has thus emerged as a potential strategy to prevent allergic diseases in infants. For example, it was proposed that the addition of specific *B. breve* strains to infant formula might assist with allergy management [98]. In summary, the administration of infant-type bifidobacteria, whether as a single species or as a synbiotic, might contribute to the prevention and treatment of allergic diseases.

### 6.3. Effects of Infant-Type Bifidobacteria on T1D and Obesity

In the context of immune-mediated diseases, the gut microbial community of infants is emerging as a critical early-life factor that is capable of influencing long-term health outcomes, such as T1D and obesity. Recent evidence suggests that an imbalance of gut microbiota composition is highly related to the pathogenesis of insulin dysfunction and T1D [99,100]. Although the exact pathogenesis of T1D is not completely clear, maladjustments in the gut microbiota composition and intestinal barrier properties have been documented in T1D subjects [99,100]. Recently, SINT1A, the Global Platform for the Prevention of Autoimmune Diabetes, investigated whether the daily administration of *B. longum* subsp. *infantis* reduces the cumulative incidence of β-cell autoantibodies in children who have a high genetic risk of developing T1D [18]. Although the study results are not yet known, it is believed that this approach holds much therapeutic potential.

Furthermore, modulation of the gut microflora associated with early rapid weight gain has been proposed as a potential strategy for curbing the growing global obesity epidemic, as evidence suggests that childhood and adult obesity is significantly associated with excessive prenatal and infant weight gain [101]. Recent studies have emphasized the potential role of bifidobacteria in obesity in early life [101,102,103]. A longitudinal study showed that normal-weight children had a higher abundance of *Bifidobacterium* in their stool samples from infancy than overweight children, who instead had a higher abundance of *Staphylococcus aureus* in their stool samples from infancy [101]. Other studies have also suggested that the abundance of bifidobacteria is inversely related to plasma glucose tolerance level, body mass index and lipopolysaccharide concentration [103]. Therefore, regulation of the intestinal microbiota via the supplementation of infant-type bifidobacteria in early life might be a strategy for the prevention of immune-mediated disorders.

## 7. Conclusions

The first 1000 days of life is a critical window for immune development and a crucial period during which the initial large-scale colonization of the gut flora of newborns occurs. Over the past 100 years, modern practices have been developed and widely applied for delivering and feeding babies. These deviations from the natural delivery and feeding processes might affect infant microbiome acquisition, particularly the bifidobacterial colonization of the gut, which is increasingly seen as a potential risk factor for immune diseases such as allergies. The gut *Bifidobacterium* population can be broadly divided into infant type (i.e., predominant in early life) and adult type (i.e., predominant in adult life). The physicochemical properties of HMOs in breast milk-fed infants confer a strong selective advantage for the gut colonization of infant-type *Bifidobacterium* species. This finding further underscores the importance of age-appropriate probiotics in the development of the immune system in the first 1000 days of life. Despite much research, it remains unclear how intestinal infant-type bifidobacteria mediate immunity in early life. Infant-type bifidobacteria not only enhance neonatal immunity by establishing important short-term protection against pathogenic infection but also provide long-term metabolic and immunological benefits. Thus, increasing the abundance of intestinal infant-type bifidobacteria has been proposed as a promising early-life health-development strategy. There is sufficient evidence to support the administration of infant-type *Bifidobacterium* strains to children with immune-mediated diseases. However, further studies are required to investigate the mechanism by which infant-type bifidobacteria influence immune system development in early life and to explore this keystone taxon and its critical ecological functions that affect the short- and long-term health outcomes of infants.

## 8. Outlook: How Do We Accelerate the Colonization of Infant-Type Bifidobacteria in the Intestinal Flora of Infants during Early Life?

An increasing body of research has investigated the role of the infant gut microbiome in long-term health outcomes throughout individuals’ lifetimes. The link between intestinal infant-type bifidobacteria and early health has been established, but the causative direction of this relationship remains unknown. Moreover, due to the limitations of the current metagenomics approaches used to evaluate the detailed compositions of complex intestinal communities, the actual contribution of infant-type bifidobacteria to the biodiversity of infant intestinal microbiota might be underestimated. Therefore, it is critical to developing optimized protocols to determine the composition of the infant gut microbiota and assess the actual contribution of infant-type bifidobacteria to early-life health and disease. Furthermore, fetal life might not be as devoid of environmental stimuli as previously thought, suggesting that infant-type bifidobacteria supplementation, both to mothers during pregnancy and to newborns in early life, might help to develop the immunity of infants. Nevertheless, several parameters of infant-type bifidobacterial supplementation remain to be determined, e.g., the optimal strain(s), single strain or synbiotic administration, and the optimal dosage and treatment duration. It is hoped that rapid technological progress in large-scale metagenomic screening will enable deeper exploration of the early-life microbiome and, thus, soon reveal the answers to these critical questions.

## Figures and Tables

**Table 1:** Summary of studies engaging specific strains of ‘infant-type’ bifidobacteria for the treatment of immune-mediated disorders.

Type of Study	Immune-Mediated Disorders	Study Object	Study Design	Study Outcomes	Conclusions	References
Clinical trial	Pathogen infection	Healthy infants at 6 to 15 weeks of age	The association of *Bifidobacterium* abundance in the stool with T-cell and antibody responses	Mean *Bifidobacterium* abundance ∝ of the CD4 T-cell responses Mean *Bifidobacterium* abundance ∝ plasma-specific IgG and stool-specific IgA Similar associations were seen for *B. longum* subsp. *infantis*.	The abundance of bifidobacteria in early infants might improve the protective effect of the vaccine by enhancing immune memory.	Huda, M.N. et al. [73]
	NEC	1237 newborns (both inpatients and transfer patients)	Strain: *B. longum* subsp. *infantis* Dose: 2.5 × 10^8^ CFU/day Duration: 1 year	NEC cases↓ NEC-associated fatalities↓	Infant-type bifidobacteria showed therapeutic effects on NEC.	Hoyos, A.B. et al. [78]
	Allergic diseases	A cohort of 65 Old Order Mennonite (OOM) and 39 Rochester mother-infant pairs	The gut microbiome and metabolome composition of atopic diseases in rural OOM infants and urban/suburban Rochester infants.	*B. longum* subsp. *infantis* was more abundant and prevalent in OOM infants. OOM infants had a lower risk of atopic diseases than Rochester infants.	A high rate of *B*. *longum* subsp. *infantis* colonization was found in the OOM infants at low risk of atopic diseases.	Seppo, A.E. et al. [19]
Pre-clinical study	Pathogen infection (rotavirus (simian SA-11))	Lewis pups	Strain: *B. breve* M-16V Dose: 1 × 10^9^ CFU/100 g/day Duration: days 2–14	Several clinical variables of severity↓ Incidence of diarrhea↓	*B. breve* M-16V seemed to be a very effective probiotic strain in ameliorating and preventing RV-induced diarrhea in children.	Azagra-Boronat, I. et al. [79]
	Pathogen infection (rhesus rotavirus)	Balb/c pups	Strain: *B. bifidum* ATCC 15696 and *B. longum* subsp. *infantis* ATCC 15697 Dose: 7.5 × 10^7^ CFU/mL (10 μL/20 μL/40 μL, increasing with age) Duration: 7 weeks	The onset and early resolution of diarrhea were observed. Rotavirus-specific IgA was elevated 16-fold in feces and 4-fold in serum.	Infant-type bifidobacteria might act as an adjuvant to alleviate the severity of diarrhea caused by rotavirus by regulating early mucous membrane and strong humoral rotavirus-specific immune response.	Qiao, H.P. et al. [80]
	Pathogen infection (Wa rotavirus)	7 day-old Balb/c pups	Strain: *B. longum* SPM1205 and SPM1206 Dose: 1 × 10^9^ CFU/mL, 150–200 μL Duration: 3 days	Rotavirus replication↓ IFN-α and IFN-β levels↑ Gene expression of IFN signaling components and IFN-inducible antiviral effectors↑	*B. longum* SPM1205 and SPM1206 effectively inhibited rotavirus replication by promoting type I IFNs to regulate the immune response.	Kang, J.Y. et al. [81]
	NEC	Cesarean-section SD rats	Strain: *B. longum* subsp. *infantis* ATCC 15697 Dose: 5 × 10^6^ CFU/day Duration: 96 h	The incidence of NEC↓ The expression of IL-6, CXCL1, TNF-α, IL-23, and iNOS↓ The expression of the antimicrobial peptides Reg3b and Reg3g↓	NEC-related inflammation could be alleviated by supplementing *B*. *longum* subsp. *infantis*.	Underwood, M.A. et al. [16]
	NEC	Naturally delivered C57BL/6 newborn mice	Strain: *B. longum* subsp. *infantis* BB-02 Dose: 3 × 10^6^ CFU in 20 μL Duration: 72 h	Intestinal permeability↓ Claudin 4 and occludin localization↑ NEC incidence↓	Administration of *B*. *infantis* reduced NEC incidence, at least in part due to its barrier-preserving properties.	Bergmann, K.R. et al. [82]
	NEC	Cesarean-section SD rats	Strain: *B. breve* M-16V Dose: 6 × 10^7^ CFU/day Duration: 96 h	Pathological scores of NEC↓ Survivability↑ Inflammation↓ TLR2 expression↑	*B. breve* M-16V prevented the development of NEC by regulating the expression of TLR and inhibiting the inflammatory response.	Satoh, T. et al. [83]
	NEC	Cesarean-section SD rats	Strain: *B. bifidum* OLB6378 Dose: 5 × 10^6^ CFU/day Duration: 96 h	The expression of lysozyme, secretory phospholipase A2, pancreatic-associated proteins 1 and 3 mRNA was elevated.	Oral administration of *B. bifidum* OLB6378 could avert both NEC and the associated increase in expression of antimicrobial peptides.	Underwood, M.A. et al. [84]
	NEC	Premature SD rats	Strain: a single strain or mixture (*B. bifidum* (PM-A0218), *B. breve* (ATCC15700), *B. longum* (PM-A0101)) Dose: 108 CFU/day Duration: 36 h	NEC↓ Mortality↓ *Escherichia coli* in stool↓ *Klebsiella* in stool↓	Administration of a mixture of *B. bifidum* and *B. longum* was most effective in preventing death and NEC.	Wu, S.-F. et al. [85]

↑ indicates promotion, ↓ indicates inhibition, and ∝ indicates a positive correlation. CFU, colony-forming units; NEC, necrotizing enterocolitis.

## Data Availability

Not applicable.
